# Factors Associated with In-Hospital Mortality in Adult Patients with Bacterial Meningitis

**DOI:** 10.3390/jcm13247845

**Published:** 2024-12-23

**Authors:** Michał Makowiecki, Marcin Paciorek, Agnieszka Bednarska, Dominika Krogulec, Dawid Porowski, Dominik Bursa, Agata Skrzat-Klapaczyńska, Carlo Bieńkowski, Justyna D. Kowalska, Magdalena Zielenkiewicz, Andrzej Horban, Tomasz Laskus

**Affiliations:** 1 Department of Adults’ Infectious Diseases, Medical University of Warsaw, 01-201 Warsaw, Polandcarlo.bienkowski@wum.edu.pl (C.B.);; 2 Institute of Mathematics, University of Warsaw, 02-097 Warsaw, Poland

**Keywords:** bacterial meningitis, SOFA, GCS, *Streptococcus pneumoniae*

## Abstract

**Background/Objectives**: The aim of this study was to evaluate the association between various clinical and laboratory findings and in-hospital mortality in community-acquired bacterial meningitis (BM). **Methods**: We retrospectively analyzed 339 adult (≥18 years old) patients with bacterial meningitis who were admitted to the Hospital for Infectious Diseases in Warsaw between January 2010 and December 2017. **Results**: Altogether, 56 patients (16.5%) died during hospitalization. On admission, the non-survivors scored lower on the Glasgow Coma Scale (GCS) (median 7 vs. 13, *p* < 0.001) and higher on the Sequential Organ Failure Assessment (SOFA) score (median 6 vs. 2, *p* < 0.001) and were less likely to complain about headaches (18.75% vs. 54.21%, *p* < 0.001) and nausea and/or vomiting (1.89% vs. 36.2%, *p* < 0.001), but were more likely to manifest peripheral nerve palsies (21.43% vs. 9.61%, *p* = 0.02). The patients who died were also more likely to be immunocompromised (53.57% vs. 34.28%, *p* = 0.01), have *Streptococcus pneumoniae* etiology (35.71% vs. 16.25%, *p* = 0.001), higher concentrations of procalcitonin (median 5.035 ng/mL vs. 2.245 ng/mL, *p* = 0.003) and urea (median 10.7 mmol/L vs. 5.865 mmol/L, *p* < 0.001) in the blood and higher protein (median 4.57 g/L vs. 2.605 g/L, *p* = 0.014) and lower glucose levels (median 0.765 mmol/L vs. 1.89 mmol/L, *p* = 0.006) in the cerebrospinal fluid (CSF). In a multiple logistic regression analysis, which was conducted separately for the GCS and SOFA, both scoring systems (OR = 0.67, OR 95% CI 0.59–0.75, *p* < 0.001 for GCS and OR = 1.42, OR 95% CI 1.29–1.60, *p* < 0.001 for SOFA) as well as an age over 70 years (OR = 3.99, OR 95% CI 1.39–12.93, *p* = 0.014) and *Streptococcus pneumoniae* etiology (OR = 2.38, OR 95% CI 1.12–4.99, *p* = 0.022) were associated with in-hospital deaths. **Conclusions**: The survivors and non-survivors with BM differed with respect to a number of signs and symptoms, etiology, the results of blood and CSF laboratory tests, and the immune deficiency status, as well as the GCS and SOFA scores. In the multiple logistic regression analysis, both of the GCS and SOFA scoring systems, age and *Streptococcus pneumoniae* etiology showed high associations with the in-hospital deaths.

## 1. Introduction

Bacterial meningitis (BM) is one of the most common infections of the central nervous system (CNS) in Europe, with an incidence ranging from 0.7 to 3.2 cases per 100,000 of the adult population per year [1,2,3,4,5,6]. In Poland, the incidence of BM in 2021 was 0.83 cases per 100,000 people, which is in the lower range for this part of the world [7]. However, the mortality of BM is high as it ranges between 10% and 23% and permanent neurological sequelae affect as many as 10–30% of survivors [1,3,8]. The mortality in BM is significantly higher than in viral meningitis (3%) but much lower than that of fungal infections with CNS involvement, which is as high as 90% for candidiasis, and 40–100% for aspergillosis [9,10].

Sequential Organ Failure Assessment (SOFA) is a widely used tool to determine the severity of organ dysfunction [11,12]. This scoring is currently a crucial part of sepsis definition as it reflects the level of multiple organ dysfunction and correlates with the outcome [11,13]. The Glasgow Coma Scale (GCS), which was introduced in 1974 and remains widely used to this day, reflects the impairment of consciousness [14,15]. While the GCS score in BM patients has been analyzed in a number of studies [1,8,16,17,18,19], SOFA (in comparison with the SAPS II, APACHE II and GCS scoring systems) has only been previously assessed in a single report [16]. However, this study included a smaller sample size of 98 Intensive Care Unit patients and, thus, the value of SOFA in predicting the outcome of BM remains unclear.

While the judicious use of antibiotics and corticosteroids has lowered the mortality and incidence of late neurological sequelae in patients with BM [20,21,22,23], the death rate remains high and it is critical to identify patients for whom current therapeutic interventions are insufficient. Previous studies have found the *Streptococcus pneumoniae* etiology [17], age [1,8,17,18,24], GCS [1,8,16,17,18,19], absence of headache [24], presence of seizures [25], temperature ≥ 38.5 °C (in patients < 65 years) [26], blood procalcitonin concentration > 7.26 ng/mL [18], thrombocytopenia [8,17], cerebrospinal fluid (CSF) leukocyte count [1,17], immunocompromised state [8], and APACHE II, SAPS II and SOFA scores [16] to be of some prognostic value in BM. The situation is further complicated by the fact that while in some studies, the unfavorable outcome was defined as in-hospital death [16,18,24,25], in others, it was a Glasgow Outcome Score (GOS) of 1–4 points [1,8,17,19]. The latter category includes patients who died as well as those who survived but suffered moderate to severe disabilities or remained in a persistent vegetative state [27]. The aim of the current study was to analyze the association between standard clinical and laboratory findings, etiology, as well as GCS and SOFA scores and in-hospital mortality. We found that *Streptococcus pneumoniae* etiology, an age over 70 years, and the SOFA and GCS scores were independently associated with in-hospital deaths.

## 2. Materials and Methods

**Figure 1:**
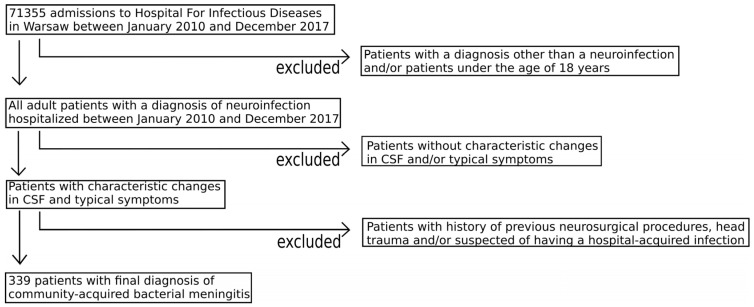
The inclusion/exclusion process of selecting patients with community-acquired bacterial meningitis.

We retrospectively analyzed demographic, clinical and laboratory data of all adult (≥18 years old) patients with a subsequent diagnosis of BM who were admitted to the Hospital for Infectious Diseases in Warsaw, Poland, between January 2010 and December 2017. There were 71,355 hospitalizations in our institution during that time. Since the aim of the study was to analyze community-acquired BM, patients who underwent neurosurgical procedures, suffered head trauma and those suspected of having a hospital-acquired infection were excluded from analysis (Figure 1). BM was diagnosed by the hospital staff physicians at the time of hospitalization. However, all electronic charts with this diagnosis were reviewed, checking for characteristic changes in the CSF (neutrophilic pleocytosis, hypoglycorrhachia, elevated protein concentration) in patients presenting with such typical symptoms and signs as headache, nausea, vomiting, fever, photophobia and nuchal rigidity [28,29,30]. Since there are no universally accepted cut-off CSF values for diagnosis of BM, none were used. The charts were reviewed independently by the primary author and two coauthors, and only once a consensus was reached that a particular patient fulfilled the above criteria for BM were their data entered into the database. The study was performed with the approval of the Bioethics Committee of the Medical University of Warsaw (case nr AKBE/145/2024). Consent for participation was not obtained, as all data were analyzed anonymously. The overwhelming majority of patients were transferred from Emergency Departments of other hospitals, where diagnostic lumbar puncture was performed and treatment was occasionally started. The etiological agents were identified by culture of CSF and blood, latex fixation tests and microscopic examination of the CSF. The GCS score was evaluated for every patient in the first minutes of presentation and SOFA score was determined based on physical examination and first laboratory tests, which were performed on admission. All patients were initially treated following current guidelines [31,32,33]. However, when the causative agent was positively identified, the patient was treated according to the antibiogram. The patients’ database was built based on charts in the years 2017–2018. The authors had access to data with which they could identify patients during data collection, but not afterwards.

Chi-square test was used to analyze nominal variables and the Mann–Whitney U-test was used to analyze continuous variables. A *p* value of ≤0.05 was considered to indicate statistical significance. Multiple logistic regression was employed to calculate the adjusted odds ratios and to determine variables independently associated with outcome. Missing data were handled by deleting the rows having null values. For the multivariate regression analysis, only records with complete data sets were included (330 out of 339 patients). Statistical analysis was performed using R ver. 3.5.2 and ver. 4.3.1-www.r-project.org (accessed on 16 December 2024).

## 3. Results

### 3.1. Clinical and Demographic Data

Altogether, 339 patients fulfilled the inclusion criteria, with 56 (16.5%) of them dying during hospitalization. The patients who died were older than the survivors (median 65 years vs. 56 years) and this difference was statistically significant (*p* < 0.01). A fatal outcome was also associated with a lower frequency of headache (18.75% vs. 54.21%, *p* < 0.001), nausea and/or vomiting (1.89% vs. 36.2%, *p* < 0.001), but with a higher prevalence of peripheral nerve palsies (21.43% vs. 9.61%, *p* = 0.02). Furthermore, the non-survivors scored lower on the GCS (median 7, IQR 6–10 vs. median 13, IQR 10–15 points, *p* < 0.001) and higher on SOFA (median 6, IQR 4–9 points vs. 2, IQR 1–4 points, *p* < 0.001). Some form of immune deficiency was present in 30 patients (53.57%) who died and in 97 survivors (34.28%) and this difference reached statistical significance (*p* = 0.01). The clinical and demographic data of all 339 patients are presented in Table 1.

### 3.2. Etiology

The data on BM etiology are presented in Table 2. *Streptococcus pneumoniae* was the most commonly identified etiological factor in both groups but was twice as frequent in the patients who died (35.71% vs. 16.25%, *p* = 0.001), while the opposite was true for *Neisseria meningitidis*, as it was less frequent in the non-survivors than in the survivors (0% vs. 10.6%, *p* = 0.02). The infecting pathogen remained undetermined in 103 (36.4%) survivors and in 16 (28.6%) fatal outcome cases.

### 3.3. Blood and Cerebrospinal Fluid

The analysis of the blood test results (Table 3) revealed that the patients who died had a higher concentration of procalcitonin (median 5.035 ng/mL, IQR 1.898–17.825 vs. median 2.245 ng/mL, IQR 0.295–11.825, *p* = 0.003) and urea (median 10.7 mmol/L, IQR 5.62–16.17 vs. median 5.865 mmol/L, IQR 4.258–8.718, *p* < 0.001) in their blood. When the CSF was analyzed (Table 3), the non-survivors were found to have higher protein concentrations (median 4.57 g/L, IQR 1.835–9.055 vs. median 2.605 g/L, IQR 1.323–5.923, *p* = 0.014) and lower glucose concentrations (median 0.765 mmol/L, IQR 0–2.24 vs. median 1.89 mmol/L, IQR 0.35–3.49, *p* = 0.006) than the survivors.

### 3.4. Multiple Logistic Regression

The factors included in the multivariate analysis were gender, age groups, GCS, SOFA and *Streptococcus pneumoniae* etiology. Since the GCS and SOFA scoring systems overlap, the analysis was performed separately for each. In the first analysis, only the GCS and age > 70 years were independently associated with in-hospital death (Table 4).

In the second analysis, which included SOFA instead of the GCS, only SOFA and *Streptococcus pneuomoniae* etiology were independently associated with a fatal outcome (Table 5).

## 4. Discussion

The current study is the largest analysis of hospitalized community-acquired BM patients in Central Europe. The mortality rate among the 339 patients was 16.5%, which is lower compared to the study of 696 patients in the Netherlands, in which the mortality of community-acquired BM was 21% [17]. The mortality rate was even higher (23%) in a retrospective study of 71 cases conducted in Japan [8], but it was only 14.3% in the study conducted by Thigpen MC et al. [34] in the USA. The relatively low mortality in our study might be related to the small number of cases due to *Streptococcus pneumoniae*, as we identified these bacteria in only 19% of patients, while in the abovementioned Netherlands study, this pathogen was responsible for 51% of the cases. However, only patients with a known etiology were included in the latter analysis and using such criteria, the prevalence of *Streptococcus pneumoniae* would increase to 30% among our patients.

*Streptococcus pneumoniae* is usually the most common etiological factor in patients with BM [1,3,6], including the elderly [35,36] and it carries a worse prognosis than other common pathogens. In our study, it was twice as frequent in the patients who died versus those who survived (35.71% vs. 16.25%). In the study by van de Beek D et al. [17], the risk of an unfavorable outcome, defined as a GOS of 1–4 points, was six times higher in the patients with *S. pneumoniae* than in the patients with *N. meningitidis*. Infection with the latter carries a good prognosis: among 150 *N. meningitidis* BM cases identified by Bijlsma MW et al. [1], the fatality rate was only 3%. In line with these observations, no deaths were recorded among our 30 patients with this etiology.

In a cohort study of patients >16 years of age by Bijlsma MW et al. [1], the age group 40–70 years (*p* = 0.041) and the age group >70 years (*p* < 0.0001) were found to be factors independently associated with an unfavorable outcome, defined as a GOS of 1–4. Several other studies found age to be associated with negative outcomes defined either by a GOS of 1–4 [8,17] or death [18,24], but different age groups were not evaluated.

The value of the GCS for predicting the outcome of BM was previously reported in some other studies. In a retrospective study by Park BS et al. [18], a multivariate analysis showed a significantly lower GCS score on admission in fatalities. Similarly, a low GCS score (less than 10 points) on admission was a predictor of an unfavorable outcome (defined as a GOS of 1–4 points) in the large study by van de Beek D et al. [17]. A lower GCS score was also independently associated with unfavorable outcomes (GOS 1–4 points) in the study published by Bijlsma MW et al. [1]. Three more groups reported similar findings: Ishihara M et al. [8] found a low GCS score to be a significant prognostic factor of an unfavorable outcome (GOS of 1–4 points), Pietraszek-Grzywaczewska I et al. [16] found a low scoring to be associated with death, while in the study by Lucas MJ et al. [19], a GCS value of 3 points was associated with high mortality. In yet another study, a decreased level of consciousness on admission was a risk factor for death, but the authors did not report the results of the GCS evaluation [25].

The SOFA score, which was an independent risk factor for in-hospital deaths in our study, has been rarely evaluated as a prognostic tool in patients with BM. Pietraszek-Grzywaczewska I et al. [16] compared four different scoring systems, APACHE II, SAPS II, GCS and SOFA, while SAPS II turned out to be the most accurate, SOFA was also a significant predictor of fatal outcome.

In our analysis, the survivors and non-survivors differed with respect to a number of clinical parameters. The patients who died were less likely to present with headache and nausea/vomiting. Similar findings were reported by McMillan DA et al. [24], who found that headache was present in 33.8% of patients who died and in 60.8% of those who survived. However, in the large prospective cohort studies by Bijlsma MW et al. [1] and by van de Beek D et al. [17], headache and nausea did not correlate with outcome. While seizures and fever were of no statistical significance in our study, Long F et al. [26] found that a temperature of ≥38.5 °C was an independent risk factor for a poor prognosis among their 181 patients, and Hussein AS and Shafran SD [25] found seizures to be a risk factor of in-hospital death.

Regarding standard laboratory tests, the procalcitonin and urea concentrations were higher in the non-survivors than in the survivors. This is in line with the study by Park BS et al. [18], in which a procalcitonin concentration > 7.26 ng/mL was an independent risk factor for death. Two other groups found an association between thrombocytopenia and an unfavorable outcome [8,17], but this was not the case in our patients.

When the CSF parameters were analyzed, the non-survivors were found to have a higher protein and lower glucose concentration, which was not the case in some other studies [1,8,17,18]. Furthermore, we found the CSF leukocyte count to be of no prognostic value, which stands in contrast to the findings reported by Bijlsma MW et al. [1] and van de Beek D et al. [17], but is consistent with the results of the study conducted by Ishihara M et al. [8]. The reasons behind these discrepancies are unclear but could be related to differences in the patient populations, infecting pathogens and/or even the definition of the unfavorable outcome itself, as it was defined as in-hospital death in our study and a low GOS in some other reports [1,8,17,19].

The mortality rate among our immunocompromised patients was twice as high as that among the non-immunocompromised patients (23.62% vs. 12.26%), which is not surprising, as immune deficiencies are known to increase the severity of infections [37,38]. The effect of immunosuppression on the BM outcome was assessed in two previous studies: van de Beek D et al. [17] analyzed 696 episodes of community-acquired meningitis, 114 of which were in immunocompromised patients, and found that immunosuppression was more frequent in patients with the unfavorable outcome, defined as a GOS of 1–4 points (27% vs. 11%), although this difference did not reach statistical significance. In this analysis, an immunocompromised state was defined as therapy with immunosuppressive drugs, diabetes mellitus, alcoholism, HIV infection and previous splenectomy. In the study by Ishihara M et al. [8], an immunocompromised state (defined as the presence of alcoholism, malignant neoplasms, diabetes mellitus, immunosuppressive therapy, terminal renal failure, liver cirrhosis and splenectomy) was associated with the poor outcome defined as a GOS of 1–4 points (52% vs. 83%). However, the latter study included only 71 patients.

Our study has several shortcomings. First, we evaluated only in-hospital mortality as the patients were not followed up with after discharge. However, such a late mortality is likely to be low. Second, antibiotic therapy was often initiated before admission at the Emergency Departments of other hospitals or occasionally by General Practitioners. However, in the former case, the treatment followed established guidelines and the patients were transferred promptly once a lumbar puncture confirmed their diagnosis. This could have obviously lowered the chance of positive pathogen identification. Another shortcoming is that the vaccination status of the patients was not known. However, at the time of the study, vaccination against *Streptococcus pneumoniae* and *Neisseria meningitidis* was rare in Poland and thus unlikely to have influenced the results. It was reported that vaccination against Pneumococcus in Poland was offered by GPs to only 10% of adult patients with chronic diseases [39]. There is also the question regarding the reliability of data on such symptoms as headache and nausea, since a large proportion of patients had decreased consciousness at admission. It is unclear whether this was fully remedied by obtaining information from families and/or asking the patient after their condition improved.

## 5. Conclusions

In conclusion, we found that the survivors and non-survivors with BM differed with respect to a number of signs and symptoms, etiology, results of blood and CSF laboratory tests, and immune deficiency status as well as GCS and SOFA score. In a multiple logistic regression analysis, both the GCS and SOFA scoring systems showed similarly high associations with in-hospital deaths.

## Figures and Tables

**Table 1:** Demographic and clinical data in 339 patients with community-acquired bacterial meningitis.

Variable	Died *N* = 56	Survived *N* = 283	*p* Value
Demography			
Age (years)	65 (54–77)	56 (37.5–67)	<0.01
Male sex	66.07%	61.13%	0.59
Clinical manifestations			
GCS (points)	7 (6–10)	13 (10–15)	<0.001 *
SOFA score	6 (4–9)	2 (1–4)	<0.001 *
Headache (%)	18.75%	54.21%	<0.001 *
Nausea and/or vomiting	1.89%	36.2%	<0.001 *
Cranial nerve palsy	12.5%	5.34%	0.09
Peripheral nerve palsy	21.43%	9.61%	0.02
Fever > 37.8 degrees Celsius	77.36%	81.16%	0.65
Meningeal signs	72.22%	80.95%	0.39
Seizures	12.43%	13.83%	0.21
Cerebellar signs and symptoms	0%	5.71%	0.15
Aphasia	3.77%	9.75%	0.25
Vertebral pain	6%	10.95%	0.42
Skin rash	9.09%	6.57%	0.7
Impaired hearing	3.85%	9.42%	0.29
Impaired memory	1.92%	3.24%	0.95
Comorbidities			
Any form of immune deficiency	53.57%	34.28%	0.01
Diabetes	16.07%	22.61%	0.36
Organ transplantation	0%	0.35%	1
Lymphoma/leukemia	10.71%	4.24%	0.099
Solid tumor	8.93%	4.95%	0.39
Immunosuppression due to chemotherapy and/or corticosteroids	17.86%	12.06%	0.34
Liver cirrhosis	5.36%	2.12%	0.36
Chronic alcohol abuse	19.64%	12.1%	0.19

Data are presented as percentage (%) with the exception of age, GCS and SOFA, which are shown as median (interquartile range); * indicates significance after Bonferroni correction for multiple comparisons. GCS, Glasgow Coma Scale; SOFA, Sequential Organ Failure Assessment score.

**Table 2:** Etiology of community-acquired bacterial meningitis in 339 patients.

Etiology	Died *N* = 56	Survived *N* = 283	*p* Value
*Streptococcus pneumoniae*	35.71%	16.25%	0.001 *
*Neisseria meningitidis*	0%	10.6%	0.02
*Staphylococcus*	8.93%	12.72%	0.57
*Listeria monocytogenes*	8.93%	6.36%	0.68
*Haemophilus influenzae*	0%	1.41%	0.83
*Mycobacterium tuberculosis*	5.36%	6.01%	1
Other Gram-positive	8.93%	6.71%	0.76
Other Gram-negative	3.57%	3.53%	1
Undetermined etiology	28.57%	36.4%	0.33

Data are presented as percentage (%). * indicates significance after Bonferroni correction for multiple comparisons.

**Table 3:** Blood and cerebrospinal fluid (CSF) laboratory findings in 339 patients with community-acquired bacterial meningitis.

Variable	Died *N* = 56	Survived *N* = 283	*p* Value
Procalcitonin (ng/mL)	5.035 (1.8975–17.825)	2.245 (0.295–11.825)	0.003
Urea (mmol/L)	10.7 (5.62–16.17)	5.865 (4.2575–8.7175)	<0.001 *
Creatinine (umol/L)	70 (44.5–105.75)	68 (56–83)	0.96
CRP (mg/L)	252 (86–348)	211 (66–324)	0.11
Lactic acid (mmol/L)	1.75 (1.335–2.83)	2.025 (1.6275–2.9375)	0.15
WBC (k/uL)	15.95 (9.75–19.9)	14.15 (10.225–20.175)	0.92
Platelet count (G/L)	159 (107.2–221)	191 (143–254)	0.1
D-Dimers (ng/mL)	3000 (1271–6529)	2280 (1210–4000)	0.12
CSF cytosis (n/uL)	658 (111.2–6378.2)	1050 (259–3754)	0.34
CSF granulocyte%	90 (74.2–95.8)	87 (70–95)	0.29
CSF protein (g/L)	4.57 (1.835–9.055)	2.605 (1.3225–5.9225)	0.014
CSF glucose (mmol/L)	0.765 (0–2.24)	1.89 (0.35–3.49)	0.006
CSF lactic acid (mmol/L)	7.315 (4.065–11.435)	6.11 (3.5–10.5)	0.39

Data are presented as median (interquartile range). * indicates significance after Bonferroni correction for multiple comparisons. CRP, C-Reactive Protein; WBC, White Blood Count.

**Table 4:** Multiple logistic regression analysis of the association between GCS, sex, age, etiology and mortality in 330 patients with community-acquired bacterial meningitis.

Variable	OR	OR 95% CI	*p* Value
Male sex	1.22	0.59–2.55	0.595
Age < 40 years	ref	ref	ref
40–70 years	1.95	0.73–5.93	0.203
>70 years	3.99	1.39–12.93	0.014
*Streptococcus pneumoniae* etiology	1.34	0.61–2.84	0.454
GCS score	0.67	0.59–0.75	<0.001

OR, odds ratio; CI, Confidence Interval; GCS, Glasgow Coma Scale.

**Table 5:** Multiple logistic regression analysis of the association between SOFA, sex, age, etiology and mortality in 330 patients with community-acquired bacterial meningitis.

Variable	OR	OR 95% CI	*p* Value
Male sex	0.96	0.47–2.01	0.914
Age < 40 years	ref	ref	ref
40–70 years	1.84	0.67–5.99	0.264
>70 years	2.95	1.00–10.05	0.061
*Streptococcus pneumoniae* etiology	2.38	1.12–4.99	0.022
SOFA score	1.42	1.29–1.60	<0.001

OR, odds ratio; CI, Confidence Interval; SOFA, Sequential Organ Failure Assessment.

## Data Availability

The raw data supporting the conclusions of this article will be made available by the authors on request.
